# *Escherichia coli* Expressing EAST1 Toxin Did Not Cause an Increase of cAMP or cGMP Levels in Cells, and No Diarrhea in 5-Day Old Gnotobiotic Pigs

**DOI:** 10.1371/journal.pone.0043203

**Published:** 2012-08-15

**Authors:** Xiaosai Ruan, Scott S. Crupper, Bruce D. Schultz, Donald C. Robertson, Weiping Zhang

**Affiliations:** 1 Veterinary and Biomedical Sciences Department/The Center for Infectious Disease Research and Vaccinology, South Dakota State University, Brookings, South Dakota, United States of America; 2 Department of Biological Sciences, Emporia State University, Emporia, Kansas, United States of America; 3 School of Veterinary Medicine, Kansas State University, Manhattan, Kansas, United States of America; University of Hyderabad, India

## Abstract

**Background:**

Enterotoxigenic *Escherichia coli* (ETEC) strains are the leading bacterial cause of diarrhea to humans and farm animals. These ETEC strains produce heat-labile toxin (LT) and/or heat-stable toxins that include type I (STa), type II (STb), and enteroaggregative heat-stable toxin 1 (EAST1). LT, STa, and STb (in pigs) are proven the virulence determinants in ETEC diarrhea. However, significance of EAST1 in ETEC-associated diarrheal has not been determined, even though EAST1 is highly prevalent among ETEC strains.

**Methodology/Principal Findings:**

In this study, we constructed *E. coli* strains to express EAST1 toxin as the only toxin and studied them in cell lines and five-day old gnotobiotic piglets to determine significance of EAST1 toxin. Data from *in vitro* studies indicated that EAST1 did not stimulate an increase of intracellular cyclic AMP or GMP levels in T-84 cells or porcine cell line IPEC-J2, nor did it enhance LT or STa toxin of ETEC strains in stimulation of cAMP or cGMP in T-84 cells. In addition, 5-day old gnotobiotic pigs challenged with *E. coli* strains expressing EAST1 as the only toxin did not developed diarrhea or signs of clinical disease during 72 h post-inoculation.

**Conclusion/Significance:**

Results from this study indicated that EAST1 alone is not sufficient to cause diarrhea in five-day old gnotobiotic pigs, and suggest that EAST1 likely is not a virulence determinant in ETEC-associated diarrhea.

## Introduction

Enterotoxigenic *Escherichia coli* (ETEC) strains are the most common bacterial cause of diarrhea. Diarrhea is the second leading cause of death to children younger than 5 years and also one of the most important diseases in young farm animals [1]–[3]. The key virulence factors of ETEC in diarrhea are bacterial adhesins and enterotoxins [4]. Adhesins mediate ETEC bacteria attachment to receptors at host epithelial cells and subsequent colonization at small intestines; whereas enterotoxins disrupt fluid homeostasis in host small intestinal epithelial cells to stimulate an increase of intracellular cyclic AMP or GMP levels and to cause electrolyte-rich fluid hyper-secretion that leads to diarrhea [4]. Enterotoxins produced by ETEC strains associated with diarrhea are heat-labile toxin (LT) and heat-stable toxins (ST) that include type I (STa), type II (STb), and enteroaggregative heat-stable toxin 1 (EAST1) [4]–[6]. While LT, STb, and STa have been identified as virulence determinants in diarrhea [4], [7]–[10], virulence significance of EAST1 in ETEC-associated diarrhea is unclear [11], [12]. The lack of determination in significance of EAST1 in ETEC-associated diarrhea causes debate regarding whether this toxin should be targeted in disease prevention, thus diffusing efforts in developing effective vaccines against ETEC diarrhea.

Contradictory observation was reported regarding the role of EAST1 that could play in diarrhea. *E. coli* or enteroaggregative *E. coli* (EAEC) strains expressing EAST1 as the only toxin were reported to cause human diarrheal outbreaks in Japan and Chile [13]–[15], and a followed case-control study suggested EAST1 as a putative agent of EAEC-associated diarrheal disease [16], [17]. In addition, epidemiological and clinical studies showed that *E. coli* strains isolated form children or travelers with diarrhea were commonly EAST1 gene positive [18]–[21]. However, the EAST1 gene was also detected in *E. coli* strains isolated from healthy or asymptomatic children and adults [15]–[17], [19], [20], [22]–[25]. Similarly to the prevalence in *E. coli* isolated from humans, the EAST1 gene was commonly detected among ETEC strains isolated from pigs with neonatal or post-weaning diarrhea [5], [6], [25]–[31], but was also found highly prevalent in *E. coli* strains isolated from pigs showing no signs of diarrhea [28]. There was one study reported that some newborn gnotobiotic piglets developed diarrhea after being inoculated with EAST1-positive EAEC strains, but clinical outcomes among the challenged piglets varied substantially and remained rather not conclusively [32],and results form that solitary pig study have never been verified [12].

Although it was initially detected among EAEC strains, EAST1 is highly prevalent among enterohemorrhagic *E. coli* (EHEC), ETEC and enteropathogenic *E. coli* (EPEC) strains [23], [33]–[37]. A recent study reported that 35% of *E. coli* strains isolated from young pigs with diarrhea carried the EAST1 gene alone or together with other toxin genes [6]. However, the role EAST1 playing in diarrhea caused by EAST1-positive ETEC, EHEC or EPEC strains has not been determined. There is evidence that porcine ETEC strains trend acquiring additional virulence factors [29]. Whether EAST1 plays a synergistic role to LT and other ST toxins of ETEC strains in virulence is also unknown.

Significance of EAST1 in diarrhea, especially ETEC-associated diarrhea, needs to be determined. EAST1 antigen has to be included as an ETEC vaccine component if EAST1 is a virulence determinant in ETEC-associated diarrhea. In this study, we cloned the EAST1 gene and constructed recombinant *E. coli* strains to express EAST1 as the only toxin, and examined the virulence significance of EAST1 in cell lines and a pig challenge model. In addition, we examined whether EAST1 enhances LT or STa toxin of ETEC strains in stimulating intracellular cAMP and cGMP and plays a synergistic role in ETEC associated diarrhea.

## Materials and Methods

### Bacterial Strains and Plasmids

Bacterial strains and plasmids used in this study are listed in Table 1. Total genomic DNA from porcine ETEC field isolate 3030-2 (K88ac/LT/STb/EAST1) was used as templates in PCR to amplify the *astA* gene encoding EAST1. Non-pathogenic porcine *E. coli* field isolate G58-1 [38], which does not carry any known toxin or fimbrial adhesin genes but can harbor plasmids to express and secrete fimbriae and toxins including LT and STa *in vitro* and *in vivo*, was used to construct *E. coli* recombinant strains carrying different EAST1-positive plasmids. Three vectors, the single-copy pBelo-BAC, medium-copy pBR322 and high-copy pUC19, were used to clone and express the *astA* gene. Enteroaggregative *E. coli* isolate 17-2 that naturally carries the EAST1 gene was transformed to express an additional LT or STa toxin, or K88ac fimbriae. Constructed strains were cultured in LB or 4AA medium supplemented with ampicillin (100 µg/ml), chloramphenicol (20 µg/ml), or tetracycline (12 µg/ml).

**Table 1:** *E. coli* strains and plasmids used in this study.

Strains and plasmids	Relevant properties	Reference
strains		
3030-2	porcine ETEC isolate, K88ac/LT/STb/EAST1	38
G58-1	porcine nonpathogenic *E. coli* isolate	38
8231	pK88ac in G58-1	this study
8321	pUC:EAST1 in 8231	this study
8322	pBR:EAST1 in 8231	this study
8323	pBelo:EAST1 in 8231	this study
17-2	EAST1+ *E. coli* field isolate	13
8722	pK88ac in 17-2	this study
8724	p8607 in 17-2	this study
8725	p8295 in 17-2	this study
TOP10	F^−^ *mcr*AΔ(*mrr*-*hsd*RMS-*mcr*BC) Φ80*lac*ZΔM15 Δ*lac*X74 *rec*A1 *deo*R*ara*D139Δ (*ara*-*leu*)7697 *gal*U *gal*K *rps*L (Str^R^)*end*A1 *nup*G	Invitrogen
8295	p8295 (STa) in TOP 10	this study
8607	p8607 (LT) in TOP 10	this study
plasmids		
pK88ac	K88ac fimbrial operon in pACYC177	this study
pUC:EAST1	*astA* gene coding EAST1 in pUC19	this study
pBR:EAST1	*astA* gene coding EAST1 in pBR322	this study
pBelo:EAST1	*astA* gene coding EAST1 in pBelo-BAC11	this study
p8295	*estA* gene coding porcine STa in pBR322	this study
p8607	*eltAB* genes coding porcine LT in pBR322	this study

### EAST1 Gene Cloning

To determine whether gene copy-number correlates to virulence, this *astA* gene was cloned into different expression vectors. The *astA* gene that was PCR amplified with primers pUC:EAST1-F (5′-atggcctgaaaagcttccggatg-‘3) and pUC:EAST1-R (5′-tccgtgggatcctgataaatcgct-‘3) was cloned into pUC19 at the HindIII and BamHI sites, with primers pBR:EAST1-F (5′-atatcctcatcgctagcgtg-‘3) and pBR:EAST1-R (5′- gcctgctggcatgcctcttc-‘3) was cloned into pBR322 at the NheI and SphI sites, and with primers pBelo:EAST1-F (5′-atggcctgaaaagcttccggatg-‘3) and pBelo:EAST1-R (5′- gcctgctggcatgcctcttc-‘3) was cloned into pBelo-BAC11 at the HindIII and SphI sites, respectively. The amplified *astA* gene products were separated with gel electrophoresis and purified with a QIAGEN gel extraction kit (QIAGEN, Valencia, CA), digested with restriction enzymes, and cloned into pUC19, pBR322, and pBelo-BAC vectors with T4 ligase (New England Biolabs, Ipswich, MA), respectively. The avirulent *E. coli* field isolate G58-1 was transformed to express K88ac fimbriae, and the resultant *E. coli* (G58/K88) was used as a host strain to be further transformed with *astA* gene positive plasmids for isogenic strains. These isogenic strains expressed the EAST1 gene in different copy-numbers and were used for the cell line and pig challenge studies. Purification, digestion, cloning and transformation were conducted by following standard protocols [39]. Antibiotics selected transformates were DNA sequenced to verify the cloned *astA* gene in correct reading frame.

### Quantitative PCR to Detect *astA* mRNA

Total mRNA from each recombinant strain was extracted using an RNeasy Mini Kit (QIAGEN). An equal amount of mRNA extracted from each strain was used in RT-PCR with the GeneAmp RNA PCR core kit (Applied Biosystems, Foster City, CA). Five microliters of each RT-PCR product were used in quantitative PCR (in triplicate) using a TaqMan probe (5′-FAM-ccgcatccagttatgcatcgtgcatatggtg-TAMRA-‘3), PCR primers astA-F (5′-atgccatcaacacagtatatc-‘3) and astA-R (5′-tcaggtcgcgagtgacgg-‘3), and gold-*taq* DNA polymerase (Applied Biosystems) in a Mx3000 QPCR system (Stratagene, La Jolla, CA) or a SmartCycler system (Cepheid, Sunnyvale, CA).

### EAST1 Competitive ELISA to Measure Expressed EAST1 Proteins

EAST1 competitive ELISA, which was developed by Robertson laboratory, was used to examine expression of EAST1 proteins from the constructed strains. Synthetic EAST1, with an average mass of 4109 (±0.25%) and a purity over 90% (Pi proteomics, Huntsville, AL) was chemically conjugated to ovalbumin (Sigma). Ten nanogram EAST1-ovalbumin conjugates (from Crupper laboratory) were used to coat each well of a microtiter plate (Costar Cat. #2595; Corning, Corning, NY) and incubated at 37°C overnight. Coated plates were blocked with 2.5% casein buffer at 37°C for 2 h. Seventy-five microliters of culture supernatants, which were resulted from centrifugation of overnight grown culture (LB or 4AA) with equal amounts of cells from each strain, were mixed with 75 µl of anti-EAST1- chicken serum (1∶1000 dilution; GenWay Biotech, Inc., San Diego, CA), and the 150 µl mixture was added to each well and further incubated at 37°C for 1 h. The plate was washed with STa ELISA washing buffer [10], and incubated with horseradish peroxidase (HRP)-conjugated donkey anti-chicken IgY (1∶2500; GenWay Biotech, Inc.). Optical density (OD) values were measured at 405 nm wavelength by a plate reader, after 20 min of reaction in a peroxidase substrate (KPL, Gaithersburg, MD).

### cAMP and cGMP ELISAs to Measure Enterotoxicity of EAST1

Enterotoxicity of LT and STa of ETEC strains can be measured through stimulation of intracellular cyclic AMP or cGMP in epithelial cells. To determine whether EAST1 possesses similar biological enterotoxicity, we examined field strain 17-2 (EAST1) and EAST1-positive recombinant strains for stimulation of cAMP or cGMP levels in human cell line T-84 (ATCC, CCL-248™) and porcine epithelial cells line IPEC-J2 (a gift from Dr. Anthony Blikslager at North Carolina State University, Raleigh, NC) using an EIA cAMP ELISA kit and an EIA cGMP ELISA kit (Assay Designs, Ann Arbor, MI). Overnight grown cultures containing equal amounts of cells, which was calculated based on culture OD values, propagated in 4AA medium from each strain were centrifuged at 3500 rpm for 30 min. One hundred and fifty microliters of resultant supernatants were added to each well containing 1–2×10^5^ T-84 cells or the IPEC-J2 cells. After 1 h incubation, intracellular cAMP and cGMP concentrations in the T-84 or IPEC-J2 cells were measured in cAMP and cGMP ELISA (acetylated version) as described previously [40], [41]. CT (cholera toxin; Sigma) and purified STa toxin (D. Robertson Laboratory) were used as positive controls.

### Ussing Chamber Assay

Enterotoxicity of EAST1 expressed by the recombinant strains was also examined in Ussing chamber assay. Overnight culture growth of each strain was centrifuged, and 2 milliliters of each supernatant were added to chambers containing monolayers of IPEC-J2 cells or T-84 cells. Short-circuit current (*I*_sc_), which represents the algebraic sum of active ion-transport processes of the cells, were measured using a modified Ussing chamber (model DCV9, Navicyte, San Diego, CA) as described previously [36]. Monolayers were bathed symmetrically with Ringer solution (in mM: 120 NaCl, 25 NaHCO_3_, 3.3 KH_2_PO_4_, 0.8 K_2_HPO_4_, 1.2 MgCl_2_, and 1.2 CaCl_2_) that was freshly prepared, maintained at 37°C, and bubbled with 5% CO_2_/95% O_2_ to maintain pH and to provide mixing. Monolayers were clamped to 0 mV using a voltage clamp apparatus (model 558C-5, University of Iowa, Dept. of Bioengineering, Iowa City, IA). A 5 s bipolar pulse was applied every 100 s and current deflections were recorded. Data acquisition was performed at 1 Hz with an Intel based computer using Aqknowledge software (v. 3.2.6, BIOPAC Systems, Santa Barbara, CA) and MP100A-CE interface.

### Gnotobiotic Piglet Challenge Study

K88ac receptor-positive piglets delivered by Cesarean-section and raised under germ-free conditions were used in the challenge study. Fecal swap samples were collected daily to monitor sterilization. At day 5, a group of 4 piglets were orally inoculated with 3×10^9^ CFUs of strain 17-2/K88ac, and another group of 4 piglets were challenged with 3×10^9^ CFUs of the G58-1/K88ac/pUC:EAST1 strain. A third group of 4 piglets without inoculation was served as the control. Inoculated piglets were observed every 3–4 hours for clinical disease, such as vomiting, diarrhea, dehydration and lethargy, and were necropsied at 72 h post-inoculation.

Pig small intestinal samples collected at necropsy were used to measure bacteria colonization using quantitative colonization assay [9], and to prepare brush border vesicles to confirm experimental piglets for expression of K88ac receptors [42]. Briefly, inside a biosafety hood, the collected ileal segment of small intestines from each piglet was cut open longitudinally and gently rinsed in cold PBS to wash off remaining feces. Rinsed ileal tissue was grinded manually in PBS (1 g tissue in 9 ml PBS) using a Pyrex glass grinder (Fisher Scientific, Pittsburgh, PA), serial diluted in PBS, and plated on LB plates. Colonies were counted after overnight growth at 37°C. Ten randomly selected colonies from each ileal sample that grew on LB plates were transferred to ampicillin-supplemented plates (100 µg/ml) to grow overnight at 37°C, and also tested in PCR for the *astA* gene, to assess plasmid retention of each inoculated strain. For brush border preparation, an ileal segment from each piglet was cut open, rinsed with hypertonic EDTA to remove feces, and incubated in hypertonic EDTA for 30 min on ice. Ileal tissue was ground gently in PBS-EDTA with an Overhead Stirrer grinder (Wheaton Instruments, Millville, NJ) and filtrated with glass wool to remove tissue debris. Brush border vesicles were collected by centrifugation, resuspended in PBS, and examined for adherence to K88ac fimbrial *E. coli* strains in bacterial adherence assay [9], [42]. In addition, an ileal segment was collected at necropsy from each piglet and fixed in neutral buffered Formalin. Fixed tissue was embedded, sectioned, H&E stained, and examined for bacterial colonization microscopically. The animal study complied with the Animal Welfare Act by following the 1996 National Research Council guidelines [43] and was approved and supervised by a state veterinarian and South Dakota State University’s Institutional Animal Care and Use Committee.

### Statistical Analysis

Data were analyzed by using the mixed procedure (SAS for windows, version 8; SAS Institute, Cary, N.C.), adjusted for multiple comparison by Bonferroni. Results were expressed as means ± standard deviations. Student’s *t*-test was used to compare different treatment groups. Calculated p values of <0.05 were regarded as significant when treatments were compared at two-tailed distribution and two-sample equal or unequal variance. In addition, the means procedure (H_0_:r = 1; H_1_:r≠1) was used to analyze data from multiple independent experiments to test whether EAST1 had significant synergistic effect on LT and STa toxins in stimulation of intracellular cAMP and cGMP levels.

## Results

### Recombinant *E. Coli* Strains were Constructed

Nine *E. coli* strains were constructed in this study (Table 1). The *astA* gene, which was isolated from the porcine ETEC strain 3030-2 and had identical DNA sequence to the *astA* gene of EAEC prototype strain O42, was cloned in the high-copy vector pUC19, the medium-copy vector pBR322, and the single-copy vector pBelo-BAC11. Resultant plasmids were used to transform an avirulent K88 fimbrial *E. coli* strain 8231for recombinant strains 8321, 8322 and 8323. The initial purpose to construct these three strains was to examine whether *astA* gene copy numbers or EAST1production levels dictate virulence significance of EAST1 in diarrhea. Strain 8321 was generated from transformation of the avirulent K88 fimbrial *E. coli* strain to express EAST1 (in pUC19 vector), and strain 8722 of field isolate 17-2 to express K88ac fimbriae. These two strains were used in the piglet challenge study to examine whether an *E. coli* strain expressing EAST1 as the only toxin is sufficiently virulent to cause diarrhea in neonatal piglets. Strains 8724 and 8725 were derived from transformation of strain 17-2 with LT plasmid p8607 and STa plasmid p8295 to express additional LT and STa, respectively, and were used for comparative studies in EIA ELISAs to determine whether EAST1 plays a synergistic role to LT or STa toxin in stimulating intracellular cAMP and cGMP *in vitro*.

### Different *astA* Gene Copy Numbers were Detected Among Recombinant EAST1 Strains

An equal amount of total mRNA from 17-2, 8722(17-2/K88ac), 8321(pUC:EAST1/K88ac), 8322(pBR:EAST1/K88ac), or strain 8323(pBelo:EAST1/K88ac) was used in RT-PCR and quantitative PCR. Quantitative PCR showed the ΔCT values of the amplified *astA* gene from strains17-2 and 8722 (17-2/K88ac) were 20.6±0.01 and 20.8±0.30, with no significant differences (p = 0.64). The ΔCT values of the *astA* gene from strains 8321, 8322, 8323 and 8231(-) were 12.3±0.02, 14.1±0.04, 17.3±0.11 and 32.7±0.66, respectively. That showed that yields of the *astA* gene amplicons from strains 8321, 8322, 8323 were correlated accordingly to the *astA*-gene plasmid copy numbers in these strains.

### EAST1 was Detected in Recombinant Strains via Competitive ELISA

**Figure 1:**
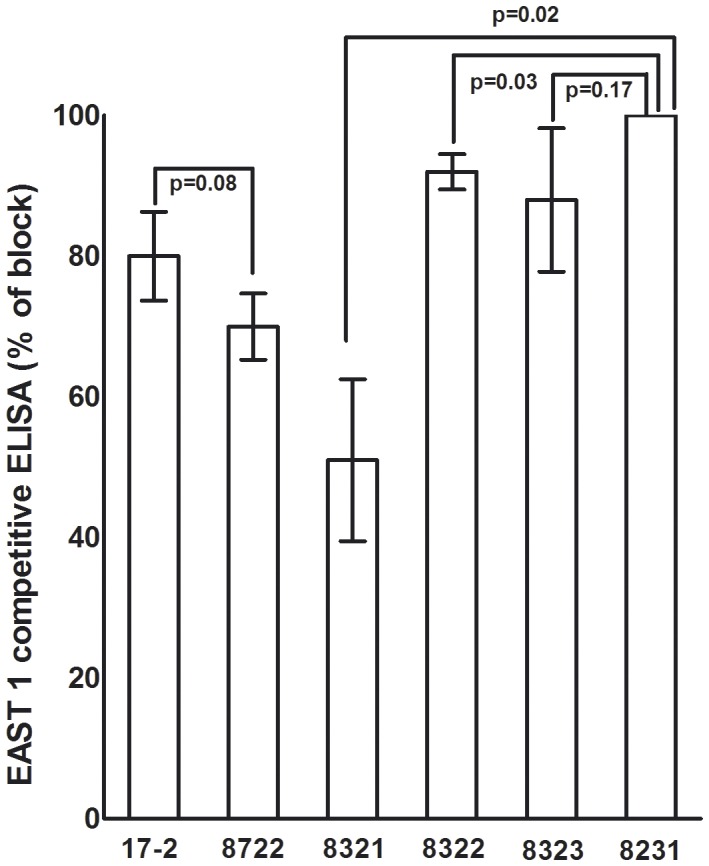
EAST1 competitive ELISA to detect EAST1protein expressed from the recombinant strains. 10 ng EAST1-ovalbumin conjugates were coated at each well of a microtiter plate at 37°C overnight. 75 µl supernatants of overnight grown culture, from an equal amount of cells, of each strain, and 75 µl of chicken anti-EAST1 serum (1∶1000; GenWay Biotech, Inc., CA) was mixed and added to each well. HRP-conjugated donkey anti-chicken IgY (1∶2500; GenWay Biotech, Inc., CA) was used as the secondary antibody. OD values were measured at 405 nm after 20 min incubation with peroxidase substrate.

EAST1 protein was detected in the overnight growth supernatant of strains 17-2, 8722, 8321 and 8322, but not from strain 8323 and the negative control strain 8231 (Fig. 1). Relative binding of anti-EAST1 antiserum to coated EAST1-ovalbumin conjugates in the wells which had culture growth supernatant of 17-2, 8722, 8321 and 8322 added was 80.7±6.4, 69.7±4.7, 51.7±11.5, and 92.3±2.5 (%), respectively. That indicated the EAST1 protein expressed by 17-2, 8722, 8321 and 8322 bound to approximate 20, 30, 48, and 8% of anti-EAST1 antiserum, respectively, and suggested the EAST1 proteins were produced and secreted by these recombinant strains.

### EAST1 Recombinant Strains did not Stimulate an Increasing of Intracellular cGMP or cAMP Levels in vitro

**Figure 2:**
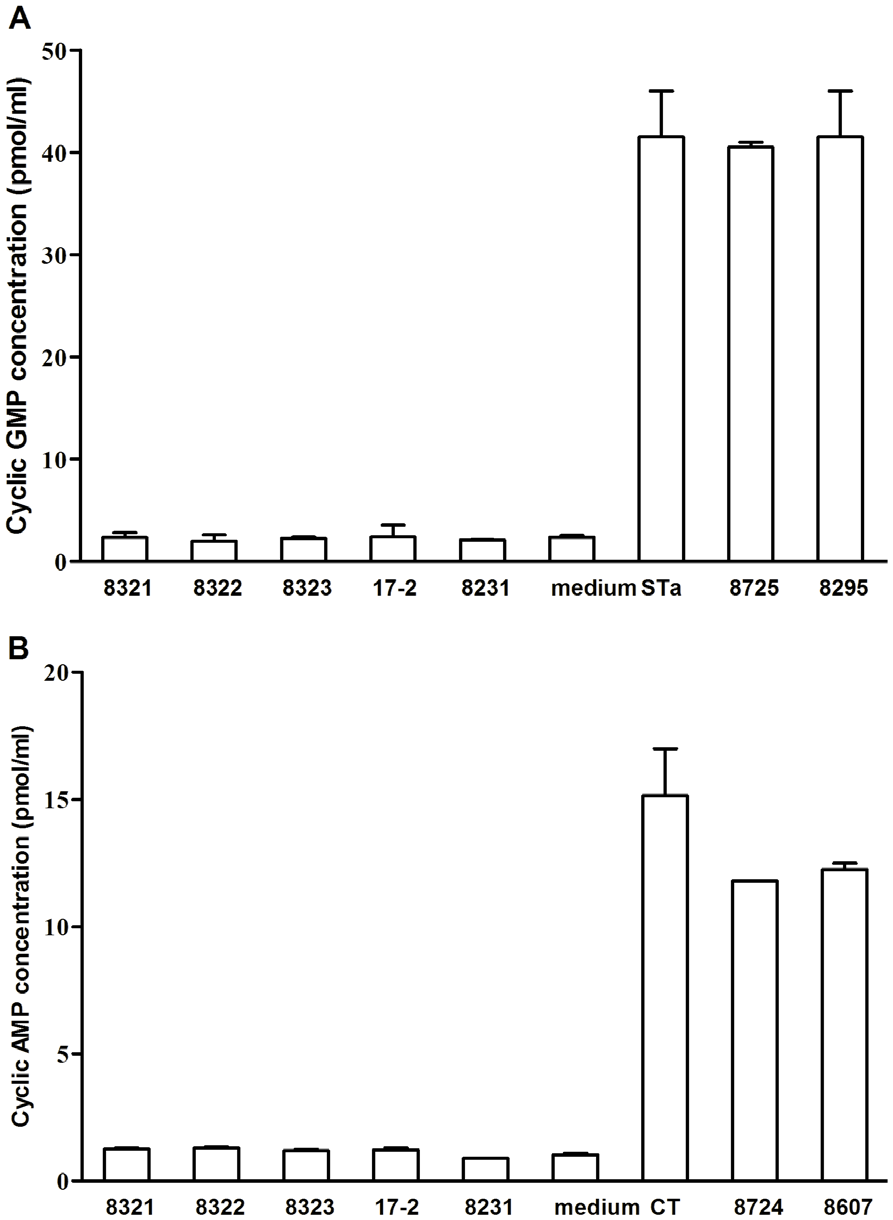
EIA ELISAs to measure stimulation of intracellular cyclic GMP and AMP in T-84 cells. Supernatants of overnight grown culture (with an equal amount of cells) from each constructed strain were incubated with 1–2×10^5^ T-84 cells. Panel A: Intracellular cGMP levels were measured using an EIA cGMP kit (Assay Designs), with 2 ng purified STa (from Robertson laboratory) was used as a positive control. Panel B: Intracellular cAMP levels were measured using an EIA cAMP kit with 10 ng CT as a positive control.

Incubation with overnight grown culture supernatant with equal amounts of 17-2, 8722, 8321 8322, or 8323 cells did not stimulate an increasing of intracellular cGMP or cAMP levels in T-84 cells (Fig. 2). The cGMP concentrations in T-84 cells incubated with supernatants of 17-2, 8722, 8321 8322, and 8323 were 2.45±1.59, 1.64±0.44, 2.33±0.67, 1.99±0.86, and 2.24±0.20 (pmole/ml), respectively. These cGMP levels were not significantly different compared to the cGMP concentration in the T-84 cells incubated with cell culture medium (2.35±0.28; p>0.05), but were significantly different compared to the cGMP in cells incubated with 2 ng STa (41.5±6.36; p<0.05) (Fig 2A). Data from the cAMP ELISA showed the cAMP concentrations in T-84 cells incubated with supernatants of strains 17-2, 8722, 8321 8322, and 8323 were 1.23±0.11, 1.32±0.12, 1.27±0.05, 1.30±0.07, and 1.20±0.07 (pmole/ml), respectively. These observed levels were not significantly different from the cAMP concentration in T-84 cells incubated with cell culture medium alone (1.04±0.08; p>0.05), but were significantly different from cAMP levels in T-84 cells incubated with 10 ng CT toxin (15.15±2.62; p<0.05) (Fig. 2B). Similarly to T-84 cells, IPEC-J2 cells showed no increases of cGMP or cAMP levels after incubation with overnight growth supernatants of strains 17-2, 8722, 8321 8322, and 8323.

**Figure 3:**
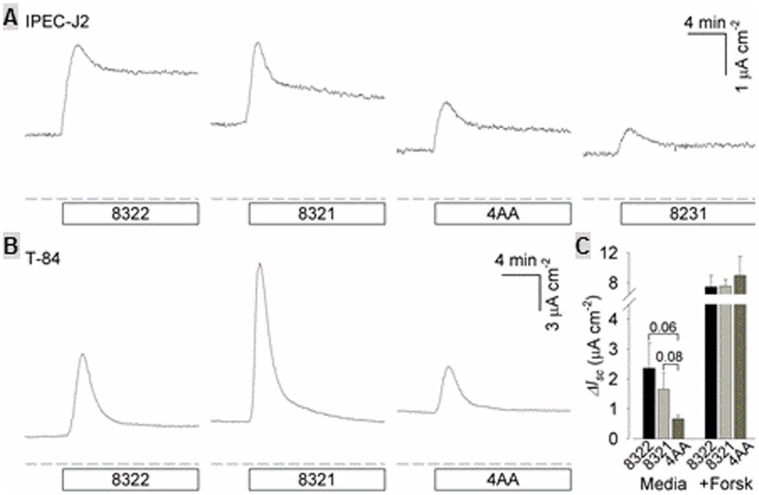
Ussing chamber assays to detect EAST1 enterotoxic activity. Panel A: Short-circuit current (*I*_sc_; µA/cm^2^) changes in monolayers of IPEC-J2 cells after incubation with culture growth of strains 8321, 8322, a negative control 8231, and 4AA medium were measured using a modified Ussing chamber. Panel B: *Isc* changes in T-84 cells incubated with culture growth of strains 8321, 8322, a negative control 8231, and 4AA medium. Panel C: The *p* values were calculated by analyzing all 5 data sets, 2 sets of data using IPEC-J2 cells and 3 sets of data using T-84 cells. Forskolin (2 µM) was added to a paired chamber as a positive control.

EAST1 expressed by the strains 8321 and 8322 did not cause significant short-circuit current (*I_sc_*) changes in Ussing chamber assay. Five independent Ussing chamber assay studies, 2 using IPEC-J2 cells and 3 with T-84 cells, were carried out. Data from these studies showed that the *I_sc_* changes were not significantly different in cells incubated with culture supernatants of 8322, 8321 or 8231 than that of 4AA culture medium. Among 5 studies, one study showed that incubation of culture growth supernatant of strains 8321 and 8322 caused *I_sc_* changes in T-84 cells and IPEC-J2 cells (Fig. 3A, 3B). But final analyses of 5 independent data sets showed *I_sc_* changes caused by strains 8321 and 8322 were not significant from that by 4AA culture medium alone (p = 0.08, p = 0.06) (Fig. 3C).

### Five-day Old Gnotobiotic Piglets did not Develop Diarrhea during 72 h Post-inoculation with 8722 and 8321 Strains

**Figure 4:**
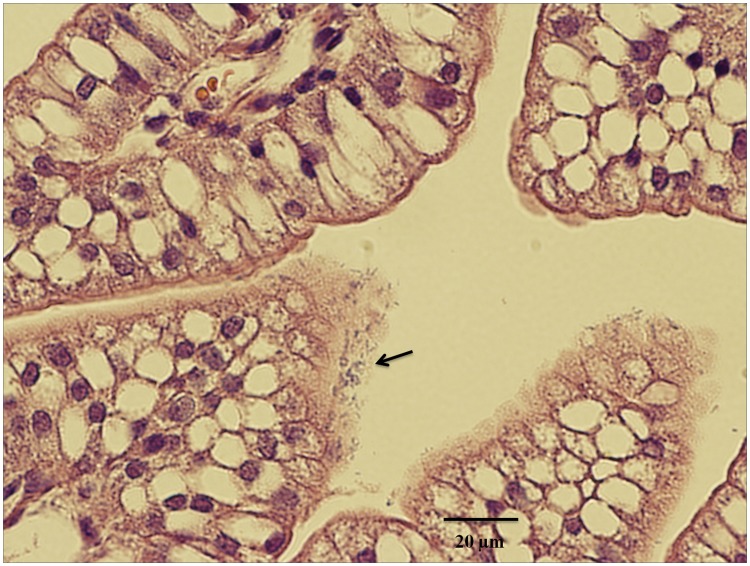
A microscopic image of piglet ileal section to show colonization of *E. coli* strain 8321 that expresses EAST1 toxin. Ileal segments of piglets challenged with strain 8321 were collected at necropsy and fixed at 10% neutral buffered Formalin. Fixed tissues were embedded, sectioned, H&E stained, and examined microscopically using an Olympus IX70 inverted microscope.

Strain 8321, that had the EAST1 gene expressed in a high-copy number expression vector pUC19, was used first to challenge 5-day old gnotobiotic piglets. If piglets challenged with strain 8321 developed diarrhea, then strains 8322 and 8323 that have the EAST1gene expressed in medium- (pBR322) and low-copy (pBelo-BAC11) vectors would be used in subsequent challenge studies. After being orally challenged with 3×10^9^ CFUs of strain 8722 or 8321, piglets remained healthy and showed no signs of diarrhea or dehydration during 72 h post-inoculation. Analyses of blood samples collected before and after inoculation showed total proteins decreased after inoculation, suggesting no dehydration occurred among inoculated piglets. To ensure that the negative outcome results were not caused by natural resistance of challenged piglets to ETEC, i.e. the lack of diarrhea in challenged piglets was resulted from their naturally resistance to ETEC colonization, we examined expression of K88ac receptors in each piglet. Bacterial adherence assays showed the brush border vesicles extracted from ileal tissue of the challenged piglets were bound by the K88ac fimbrial *E. coli* strain 8231 (16–27 bacteria per brush border vesicle). In addition, data from the quantitative colonization study showed challenged piglets were well colonized. Piglets challenged with strains 8722 and 8321 had 4.4±1.3 and 8.5±4.0 (×10^9^) CFU bacteria colonized per gram of ileal tissue at 72 h post-inoculation. Differences of colonization in piglets inoculated with these two strains were statistically not significant (p = 0.11). Colony screening indicated challenge strains had the introduced plasmids retained, as all 10 colonies (of each piglet) transferred from the LB plates grew well on ampicillin-supplemented (100 µg/ml) plates. PCR screening proved that all colonies were positive of the *astA* gene. In addition, microscopic images of ileal tissue section showed bacteria colonized at small intestinal epithelial cells of the challenged piglets (Fig. 4).

### EAST1 did not Enhance LT or STa Toxin of ETEC in Stimulation of Intracellular cAMP or cGMP In vitro

EAST1 did not act synergistically to LT or STa toxin in stimulating intracellular cAMP or cGMP in T-84 cells (Table 2). Supernatant from LB and 4AA overnight grown culture from strains 8724 (EAST1/LT) and 8607 (LT) were examined for stimulation of cAMP; whereas supernatants from 8725 (EAST1/STa) and 8295 (STa) were comparatively studied for stimulation of cGMP (Table 2). As variations were observed among studies, the test of means was used for the final analysis of all data sets. Results indicated the cGMP concentrations of T-84 cells incubated with STa alone (strain 8295) and STa/EAST1 (strain 8725) were not significantly different (p = 0.28). Similarly, no significant differences were detected in cAMP levels in T-84 cells incubated with strain 8724 that expresses LT and EAST1 and strain 8607 that expresses LT alone (p = 0.94). The cAMP or cGMP levels in T-84 cells incubated with EAST1 alone (strain 17-2) showed no differences compared to those incubated with only cell culture medium.

**Table 2:** Intracellular cyclic AMP and GMP levels (pmole/ml) in T-84 cells incubated with culture supernatant of strains 8724 (EAST1/LT), 8607 (LT), 8725 (EAST1/STa), 8295 (STa), 17-2 (EAST1), or cell culture medium, measured using cAMP or cGMP EIA kits (Assay Design).

	cyclic AMP (pmole/ml)				cyclic GMP (pmole/ml)			
	8724 (EAST1/LT)	8607 (LT)	medium	17-2 (EAST1)	8725 (EAST1/STa)	8295 (STa)	medium	17-2 (ESAT1)
LB	280, 240*	300, 300*	2.7, 2.8	ND	450, 465*	850, 940*	3.6,0.7	ND
	9.8, 10.1	11.7, 11.5	1.0, 0.9	ND	99, 56	86,98	1.1, 0.9	ND
	12.6, 13.8	20, 14	1.6, 1.8	ND	175, 133	150, 150	0.8, 0.9	ND
4AA	10.1, 11.4	9.9, 9.4	1.0, 1.0	1.2, 1.2	70, 70	56, 52	3.2, 2.4	ND
	11.8, 11.8	12.5, 12	1.3, 1.2	1.3, 1.2	19, 26	72, 64	1.7, 0.7	0.7, 2.5
	19.5, 17.5	10.6, 12.2	0.8, 1.0	1.2, 1.2	10.2, 10	16.5, 10.1	1.1, 1.1	ND
					46, 37	41, 40	2.6, 2.2	3.6,1.2

* A non-acetylated version analysis was used to measure cAMP or cGMP concentrations in initial assays. ND indicates not done.

Expression of LT in strains 8607 (LT) and 8724(LT/EAST1) was verified in a GM1 ELISA as described previously [6], [10], and expression of STa toxin from strains 8295 (STa) and 8725 (STa/EAST1) was examined in a STa competitive ELISA [10], [41]. In GM1 and STa ELISAs, 400 ng GM1 (Sigma) was coated to each well of a MaxSorb plate (Nunc, Roskilde, Denmark) and 1.25 ng STa-ovalbumin conjugates (Robertson Laboratory) to each well of a Costar plate (Corning, Inc., Corning, NY), with anti-CT (1∶3300; Sigma) and anti-STa (1∶10,000; Robertson laboratory) as the primary antibody, respectively. HRP-conjugated goat-anti-rabbit IgG (1∶2500 in GM1 and 1∶10,000 in STa ELISA) was used as the secondary antibody. GM1 ELISA data showed supernatants of overnight cultures from strains 8607 and 8724 had OD readings of 0.972±0.03 and 0.92±0.04 (p = 0.10), respectively; and the competitive STa ELISA showed the OD values for strains 8295 and 8725 were 0.177±0.02 and 0.211±0.01 (p = 0.30), respectively. These ELISA data indicated that strains 8607 (LT) and 8724 (EAST1/LT) expressed an equivalent level of LT toxin, whereas strains 8725 (EAST1/STa) and 8925 (STa) expressed a similar level of STa toxin. Therefore, if EAST1 had synergistic effects, cells incubated with 8724 would have had a higher cAMP level, and cells incubated with 8725 would have had a higher cGMP level.

## Discussion

Significance of EAST1 in diarrhea has not been determined previously. EAST1 was reported as the only toxin produced by *E. coli* strain O166 that caused an outbreak of gastroenteritis in Japan in 1996 [44], and EAST1-positive EAEC strain 17-2 that was suggested to associate with an outbreak in Chile [13]. However, EAST1 was also detected in EAEC and other types of *E. coli* strains isolated from asymptomatic or healthy individuals [15], [25]. Moreover, human volunteers inoculated with 17-2 strain did not develop diarrhea [45], that suggests EAST1 is not the sole mediator to cause human diarrhea. Similar to the occurrence in human strains, the EAST1 gene was detected among ETEC strains isolated from pigs with diarrhea [5], [6], [30], but also commonly found in *E. coli* strains isolated from healthy pigs [28]. One study reported that one-day old gnotobiotic piglets, after oral inoculation of 1–2×10^10^ CFUs of strain 17-2, 2 of 6 inoculated piglets died in 18 h, another 2 became severely ill, but the other 2 remained healthy [32]. It was unknown what caused such great variation in disease outcomes among these experimental pigs. The one-day old piglets, however, especially being inoculated with such a high dose, could be overwhelmed and develop diarrhea, even with an avirulent *E. coli* strain (D. Francis, personal communication). In the current study, we challenged 5-day old piglets with 3×10^9^ CFUs of inoculum. We believe that 5-day old piglets tolerated *E. coli* inoculation, especially at a reasonable dose, better than the newborn piglets, and could be natural in disease progression after infection.

In order to eliminate ambiguity caused by host genetic resistance, we used the K88ac fimbrial strains 8722 (17-2/K88; not 17-2) and 8321(EAST1/K88), and K88ac-receptor positive piglets in the pig challenge study. It has been demonstrated that experimental piglets would not develop disease even if being inoculated with a diarrheal ETEC strain if they lack receptors (at epithelial cells) to be attached by bacteria fimbriae or adhesins [46], [47]. Using K88ac-receptor-positive piglets in the challenge study should generate conclusive results, because these pigs are naturally susceptible to K88ac fimbrial ETEC strains and always develop diarrhea and/or dehydration after inoculation. Data from bacterial adherence assays indicated the challenged piglets were K88ac-receptor positive. In addition, the quantitative colonization study and the microscopic study revealed piglets were well colonized by the challenge strains at small intestines. To also ensure plasmids carrying EAST1 were retained by the challenge strain 8321*in vivo*, we cultured bacteria recovered from pig small intestines on LB plates and transferred 10 colonies to grow at ampicillin-supplemented plates. Data showed all colonies grew on ampicillin plates, indicating these colonies carried the plasmids that carry ampicillin resistance gene. When examined in PCR, these colonies were positive of the EAST1 gene. Future studies to culture bacteria that are isolated from ileal segments of challenged piglets simultaneously at the LB plates and antibiotics-supplemented plates (with great sampling sizes) will help us to better assess colonization of challenge strains *in vivo*. When we cultured 8321 at the LB and ampicillin-supplemented plates *in vitro*, we observed no plasmid loss occurred through three passes (2.28±0.39×10^7^ CFUs on LB vs. 2.84±0.14×10^7^ CFUs on ampicillin plates; p = 0.19). These data suggested that the EAST1 plasmids likely were stably maintained in challenge strain 8321 *in vivo* and *in vitro*. Therefore, if EAST1 were a virulence determinant in diarrhea, these challenged piglets would have developed clinic disease. Given that none of them developed any sign of diarrhea, the same as the piglets from the unchallenged negative control group, we concluded that EAST1 is unlikely a virulence determinant in porcine neonatal diarrhea. Although a positive control group was not directly included, 2 piglets from the same litter that were used in a separated project developed severe diarrhea and showed dehydration signs including backbone very prominent, skin turgor and eyes sunken in orbits, after 24 h post-inoculation with porcine ETEC strain 3030-2.

To ensure expression of the additional K88ac fimbria did not negatively affect the expression of EAST1 in strain 8722 (17-2/K88), we conducted quantitative PCR to measure *astA* gene at the mRNA level and performed a competitive ELISA to determine EAST1 expression in strains 8722 and 17-2. Results showed nearly identical ΔCT values (20.6±0.01 and 20.8±0.30; p = 0.64) from amplified EAST1 gene were obtained from quantitative PCR, and similar production of EAST1 (p = 0.08) was detected in EAST1 competitive ELISA. These data indicated expression of additional K88ac fimbriae did not significantly affect strain 17-2 in production of EAST1. Therefore, piglets challenged with 8722 strain would not develop diarrheal disease had they been challenged with the wild-type 17-2 strain, even were these piglets well colonized by 17-2 at the small intestines. We need to point out that we verified the cloned EAST1 gene was expressed and the EAST1 proteins were produced and secreted by the constructed strains *in vitro*, we were unable to verify that expression of EAST1 in the challenge strains was not affected *in vivo*, especially considering culture conditions and prolonged incubation inside the piglet guts; even though the same host strain and the same expression vectors were demonstrated to express LT and other heat-stable toxins including STa and STb equally effective in culture medium and pig small intestines [9], [48]. Unfortunately, we currently do not have optimal methods to confirm that the challenge strains express EAST1 at a similar level *in vitro* and *in vivo*. Future studies to develop methods to measure expression of EAST1 inside piglet guts, or to use an EAST1 knockout mutant strain derived from a porcine diarrheagenic *E. coli* strain that expresses EAST1 as the only toxin, were it possible, in piglet challenge studies, will further conclusively assess the role of EAST1 in porcine diarrhea disease.

It was reported that *E. coli* strains carry different copy numbers of the *astA* gene, and thus it was suggested the gene copy numbers may affect significance of EAST1 in disease [5], [23], [49]. Attempting to determine whether EAST1 production influences virulence, we cloned the *astA* gene in three different expression vectors: the single-copy pBelo-BAC11, the medium-copy pBR322, and the high-copy pUC19 and constructed strains to express *astA* mRNA and protein at the levels correlated to the copy numbers of the carried expression vectors. But as piglets did not develop diarrhea even after being challenged with strain 8321 that had EAST1cloned and expressed at the high-copy vector, we did not continue challenge studies using strains 8322 or 8323. We believed it is unlikely that strains 8322 and 8323, that had EAST1 cloned and expressed in a medium-copy or a single-copy vector, or perhaps EAST1-positive *E. coli* field isolates that carry one or a few copies of the *astA* gene, would cause diarrhea in pigs.

Purified EAST1 or culture ultrafiltrate was reported previously to cause short-circuit current changes to cell lines [36] and rabbit ileal tissue [35], but data from this study revealed differently. In this study, we used culture supernatant resulted from ultracentrifugation, which has EAST1 detected in competitive ELISA, were used to treat IPEC-J2 or T-84 cells. Pooled data (from 5 data sets) showed that the differences in *I_sc_* changes caused by strains 8321 and 8322 were not significant, even compared to those in cells incubated with the cell culture medium alone.

EAST1 is often compared to STa toxin due to similarity of a peptide domain [36], and was suspected to have similar biological functions [36], [50]. However, unlike STa, EAST1 does not stimulate fluid accumulation in suckling mice or cause diarrhea in human volunteers [45]. Although EAST1^+^ EAEC strains were linked to diarrhea, it is thought that not one set of virulence factors, but rather combinations of multiple virulence factors, are associated with diarrhea [12]. Data from this study showed that EAST1 did not stimulate an increase of intracellular cGMP or cAMP. In addition, data also showed EAST1 had no synergistic effect to LT or STa toxin of ETEC in stimulation of intracellular cAMP or cGMP *in vitro*. Since an earlier report that indicated porcine ETEC strains trend to acquire additional toxins over time [29], and the fact that EAST1 is frequently carried along with LT, STa and STb toxins by ETEC strains associated with diarrhea in pigs [5], [6], [28], [30], EAST1 has been speculated to have synergistic enterotoxicity in ETEC strains. Given ETEC strains expressing LT, STa, or sometimes STb alone are already sufficiently virulent to cause diarrhea in pigs [7], [9], [10], we questioned whether acquisition of an additional toxin makes these ETEC strains more virulent, possibly to overcome enhanced host immunity gained from natural exposure to ETEC pathogens, vaccination or antibiotic treatment. Data from this study clearly indicated that EAST1 did not enhance LT in stimulation of intracellular cAMP or STa in stimulation of intracellular cGMP in the human cell line T-84. Therefore, EAST1 is unlikely a synergistic factor to other ETEC toxins in stimulation of fluid hyper-secretion. It was also reported that EAST1 positive *E. coli*, mainly EAEC strains, cause intestinal inflammation [51], [52]. But histological examination found no apparent inflammation occurring in small intestines of the challenged piglets, suggesting EAST1 alone may not contribute significantly to intestinal inflammation. However, future studies to examine IL-8 or other cytokine levels in challenged piglets, and perhaps more important to further characterize interaction and specificity between EAST1 toxin and host receptors, may help us to better evaluate whether EAST1 positive ETEC strains manifest intestinal inflammation.

The EAST1-positive EAEC prototype strain O42 caused diarrhea in majority of human volunteers [45]. That likely contributes to the assumption that EAST1-positive strain 17-2 could also cause diarrhea and EAST1 could be a virulence determinant in diarrhea. However, recent whole-genome sequencing data showed strain O42 carries other genes that strain 17-2 does not, such as the *shETBA* gene, as well as chloramphenicol and tetracycline resistance genes [53]. In an attempt to determine whether ShET_BA_ toxin stimulates fluid secretion and causes diarrhea, we cloned the *shETBA* gene into vector pBR322 and expressed it in TOP 10 *E. coli* cells, incubated T-84 cells with supernatants of the ShET_BA_ recombinant strain, and measured cAMP and cGMP levels with ELISAs. We found no increasing of cAMP or cGMP levels in the T-84 cells under our conditions; inferring that ShET_BA_ toxin may also not play a significant role in diarrhea. However, we only verified the cloned gene with DNA sequencing but not the expression or secretion of the ShET_BA_ toxin due to lack of anti-ShET_BA_ antibodies, neither did we conduct any animal challenge studies. Observation regarding significance of ShET toxin obtained from this study could be premature. It was also found that the *astA* genes carried by strains O42 and 17-2 differ at nucleotides coding the 21^th^ amino acid. This mutation, from a more hydrophobic nonpolar alanine in strain O42 to a less hydrophobic but polar threonine in strain 17-2, was speculated to reduce EAST1 toxicity and thus virulence in strain 17-2 [45], [54]. But the *astA* gene used in this study to construct recombinant strains is identical to that of strain O42. That suggests the substitution of this particular amino acid unlikely dictates the significance of EAST1.

Data from this study suggest that EAST1 alone is not sufficient to cause diarrhea in young gnotobiotic pigs, and does not stimulate increases of intracellular cAMP or cGMP in the human colon cell line T-84 or porcine small intestinal cell line IPEC-J2. Data from this study also suggest EAST1 does not enhance LT and STa toxins in stimulating intracellular cAMP or cGMP in T-84 cells. Together, our data indicated that in the animal tested and under the test conditions used, EAST1 is not a virulence determinant in ETEC diarrhea.

## References

[1] Black RE, Cousens S, Johnson HL, Lawn JE, Rudan I, et al (2010) Global, regional, and national causes of child mortality in 2008: a systematic analysis. Lancet 375: 1969–1987. 20466419 10.1016/S0140-6736(10)60549-1

[2] WHO (2006) Future directions for research on enterotoxigenic Escherichia coli vaccines for developing countries. Wkly Epidemiol Rec 81: 97–107. 16671213

[3] USDA (2002) USDA:APHIS:VS, CEAH, Nat. Anim. Health Monitoring Syst. Part II: Reference for swine health and health management in the United States, 2000, Ft. Collins, CO.

[4] Nataro JP, Kaper JB (1998) Diarrheagenic Escherichia coli. Clinical microbiology reviews 11: 142–201. 9457432 10.1128/cmr.11.1.142 PMC121379

[5] Frydendahl K (2002) Prevalence of serogroups and virulence genes in Escherichia coli associated with postweaning diarrhoea and edema disease in pigs and a comparison of diagnostic approaches. Veterinary microbiology 85: 169–182. 11844623 10.1016/s0378-1135(01)00504-1

[6] Zhang W, Zhao M, Ruesch L, Omot A, Francis D (2007) Prevalence of virulence genes in Escherichia coli strains recently isolated from young pigs with diarrhea in the US. Veterinary microbiology 123: 145–152. 17368762 10.1016/j.vetmic.2007.02.018

[7] Berberov EM, Zhou Y, Francis DH, Scott MA, Kachman SD, et al (2004) Relative importance of heat-labile enterotoxin in the causation of severe diarrheal disease in the gnotobiotic piglet model by a strain of enterotoxigenic Escherichia coli that produces multiple enterotoxins. Infection and immunity 72: 3914–3924. 15213135 10.1128/IAI.72.7.3914-3924.2004 PMC427467

[8] Erume J, Berberov EM, Kachman SD, Scott MA, Zhou Y, et al (2008) Comparison of the contributions of heat-labile enterotoxin and heat-stable enterotoxin b to the virulence of enterotoxigenic Escherichia coli in F4ac receptor-positive young pigs. Infection and immunity 76: 3141–3149. 18426880 10.1128/IAI.01743-07 PMC2446739

[9] Zhang W, Berberov EM, Freeling J, He D, Moxley RA, et al (2006) Significance of heat-stable and heat-labile enterotoxins in porcine colibacillosis in an additive model for pathogenicity studies. Infection and immunity 74: 3107–3114. 16714538 10.1128/IAI.01338-05 PMC1479275

[10] Zhang W, Robertson DC, Zhang C, Bai W, Zhao M, et al (2008) Escherichia coli constructs expressing human or porcine enterotoxins induce identical diarrheal diseases in a piglet infection model. Applied and environmental microbiology 74: 5832–5837. 18658289 10.1128/AEM.00893-08 PMC2547035

[11] Nishikawa Y, Zhou Z, Hase A, Ogasawara J, Cheasty T, et al (2000) Relationship of genetic type of Shiga toxin to manifestation of bloody diarrhea due to enterohemorrhagic Escherichia coli serogroup O157 isolates in Osaka City, Japan. Journal of clinical microbiology 38: 2440–2442. 10835027 10.1128/jcm.38.6.2440-2442.2000 PMC86837

[12] Harrington SM, Dudley EG, Nataro JP (2006) Pathogenesis of enteroaggregative Escherichia coli infection. FEMS microbiology letters 254: 12–18. 16451173 10.1111/j.1574-6968.2005.00005.x

[13] Nataro JP, Kaper JB, Robins-Browne R, Prado V, Vial P, et al (1987) Patterns of adherence of diarrheagenic Escherichia coli to HEp-2 cells. The Pediatric infectious disease journal 6: 829–831. 3313248 10.1097/00006454-198709000-00008

[14] Tzipori S, Smith M, Birch C, Barnes G, Bishop R (1983) Cryptosporidiosis in hospital patients with gastroenteritis. The American journal of tropical medicine and hygiene 32: 931–934. 6625074 10.4269/ajtmh.1983.32.931

[15] Yamamoto T, Echeverria P (1996) Detection of the enteroaggregative Escherichia coli heat-stable enterotoxin 1 gene sequences in enterotoxigenic E. coli strains pathogenic for humans. Infection and immunity 64: 1441–1445. 8606115 10.1128/iai.64.4.1441-1445.1996 PMC173940

[16] Howell A, Harland RN, Barnes DM, Hayward E, Redford J, et al (1987) Endocrine therapy for advanced carcinoma of the breast: effect of tumor heterogeneity and site of biopsy on the predictive value of progesterone receptor estimations. Cancer research 47: 296–299. 3791214

[17] Vial PA, Robins-Browne R, Lior H, Prado V, Kaper JB, et al (1988) Characterization of enteroadherent-aggregative Escherichia coli, a putative agent of diarrheal disease. The Journal of infectious diseases 158: 70–79. 2899125 10.1093/infdis/158.1.70

[18] Bhan MK, Raj P, Levine MM, Kaper JB, Bhandari N, et al (1989) Enteroaggregative Escherichia coli associated with persistent diarrhea in a cohort of rural children in India. The Journal of infectious diseases 159: 1061–1064. 2656875 10.1093/infdis/159.6.1061

[19] Gascon J, Vargas M, Schellenberg D, Urassa H, Casals C, et al (2000) Diarrhea in children under 5 years of age from Ifakara, Tanzania: a case-control study. Journal of clinical microbiology 38: 4459–4462. 11101580 10.1128/jcm.38.12.4459-4462.2000 PMC87621

[20] Gascon J, Vargas M, Quinto L, Corachan M, Jimenez de Anta MT, et al (1998) Enteroaggregative Escherichia coli strains as a cause of traveler’s diarrhea: a case-control study. The Journal of infectious diseases 177: 1409–1412. 9593036 10.1086/517826

[21] Itoh Y, Nagano I, Kunishima M, Ezaki T (1997) Laboratory investigation of enteroaggregative Escherichia coli O untypeable:H10 associated with a massive outbreak of gastrointestinal illness. Journal of clinical microbiology 35: 2546–2550. 9316905 10.1128/jcm.35.10.2546-2550.1997 PMC230008

[22] Fujihara S, Arikawa K, Aota T, Tanaka H, Nakamura H, et al (2009) Prevalence and properties of diarrheagenic Escherichia coli among healthy individuals in Osaka City, Japan. Japanese journal of infectious diseases 62: 318–323. 19628916

[23] Savarino SJ, McVeigh A, Watson J, Cravioto A, Molina J, et al (1996) Enteroaggregative Escherichia coli heat-stable enterotoxin is not restricted to enteroaggregative E. coli. The Journal of infectious diseases 173: 1019–1022. 8603943 10.1093/infdis/173.4.1019

[24] Dougan G, Sellwood R, Maskell D, Sweeney K, Liew FY, et al (1986) In vivo properties of a cloned K88 adherence antigen determinant. Infection and immunity 52: 344–347. 2870029 10.1128/iai.52.1.344-347.1986 PMC262244

[25] Yamamoto T, Nakazawa M (1997) Detection and sequences of the enteroaggregative Escherichia coli heat-stable enterotoxin 1 gene in enterotoxigenic E. coli strains isolated from piglets and calves with diarrhea. Journal of clinical microbiology 35: 223–227. 8968912 10.1128/jcm.35.1.223-227.1997 PMC229543

[26] Choi C, Kwon D, Chae C (2001) Prevalence of the enteroaggregative Escherichia coli heat-stable enterotoxin 1 gene and its relationship with fimbrial and enterotoxin genes in E. coli isolated from diarrheic piglets. Journal of veterinary diagnostic investigation : official publication of the American Association of Veterinary Laboratory Diagnosticians, Inc 13: 26–29. 10.1177/104063870101300106 11243358

[27] Choi C, Cho W, Chung H, Jung T, Kim J, et al (2001) Prevalence of the enteroaggregative Escherichia coli heat-stable enterotoxin 1 (EAST1) gene in isolates in weaned pigs with diarrhea and/or edema disease. Veterinary microbiology 81: 65–71. 11356319 10.1016/s0378-1135(01)00332-7

[28] Ngeleka M, Pritchard J, Appleyard G, Middleton DM, Fairbrother JM (2003) Isolation and association of Escherichia coli AIDA-I/STb, rather than EAST1 pathotype, with diarrhea in piglets and antibiotic sensitivity of isolates. Journal of veterinary diagnostic investigation : official publication of the American Association of Veterinary Laboratory Diagnosticians, Inc 15: 242–252. 10.1177/104063870301500305 12735346

[29] Noamani BN, Fairbrother JM, Gyles CL (2003) Virulence genes of O149 enterotoxigenic Escherichia coli from outbreaks of postweaning diarrhea in pigs. Veterinary microbiology 97: 87–101. 14637041 10.1016/j.vetmic.2003.08.006

[30] Osek J (2003) Detection of the enteroaggregative Escherichia coli heat-stable enterotoxin 1 (EAST1) gene and its relationship with fimbrial and enterotoxin markers in E. coli isolates from pigs with diarrhoea. Veterinary microbiology 91: 65–72. 12441232 10.1016/s0378-1135(02)00262-6

[31] Veilleux S, Dubreuil JD (2006) Presence of Escherichia coli carrying the EAST1 toxin gene in farm animals. Veterinary research 37: 3–13. 16336921 10.1051/vetres:2005045

[32] Tzipori S, Montanaro J, Robins-Browne RM, Vial P, Gibson R, et al (1992) Studies with enteroaggregative Escherichia coli in the gnotobiotic piglet gastroenteritis model. Infection and immunity 60: 5302–5306. 1452364 10.1128/iai.60.12.5302-5306.1992 PMC258311

[33] Sackett DL (1980) Hypertension VI. Follow-up of the treated hypertensive patient. Can J Public Health 71: 225–230. 7407724

[34] Paiva de Sousa C, Dubreuil JD (2001) Distribution and expression of the astA gene (EAST1 toxin) in Escherichia coli and Salmonella. International journal of medical microbiology : IJMM 291: 15–20. 11403406 10.1078/1438-4221-00097

[35] Savarino SJ, Fasano A, Watson J, Martin BM, Levine MM, et al (1993) Enteroaggregative Escherichia coli heat-stable enterotoxin 1 represents another subfamily of E. coli heat-stable toxin. Proceedings of the National Academy of Sciences of the United States of America 90: 3093–3097. 8385356 10.1073/pnas.90.7.3093 PMC46243

[36] Veilleux S, Holt N, Schultz BD, Dubreuil JD (2008) Escherichia coli EAST1 toxin toxicity of variants 17-2 and O 42. Comparative immunology, microbiology and infectious diseases 31: 567–578. 10.1016/j.cimid.2007.10.003 18243316

[37] Yamamoto T, Taneike I (2000) The sequences of enterohemorrhagic Escherichia coli and Yersinia pestis that are homologous to the enteroaggregative E. coli heat-stable enterotoxin gene: cross-species transfer in evolution. FEBS letters 472: 22–26. 10781798 10.1016/s0014-5793(00)01414-9

[38] Francis DH, Willgohs JA (1991) Evaluation of a live avirulent Escherichia coli vaccine for K88+, LT+ enterotoxigenic colibacillosis in weaned pigs. American journal of veterinary research 52: 1051–1055. 1679980

[39] Kung M, Tachmes L, Birch SJ, Fernandez RJ, Abraham WM, et al (1980) Hemodynamics at rest and during exercise in comfortable, hot and cold environments. Measurement with a rebreathing technique. Bulletin europeen de physiopathologie respiratoire 16: 429–441. 7407435

[40] Zhang C, Zhang W (2010) Escherichia coli K88ac fimbriae expressing heat-labile and heat-stable (STa) toxin epitopes elicit antibodies that neutralize cholera toxin and STa toxin and inhibit adherence of K88ac fimbrial E. coli. Clinical and vaccine immunology : CVI 17: 1859–1867. 20980482 10.1128/CVI.00251-10 PMC3008177

[41] Zhang W, Zhang C, Francis DH, Fang Y, Knudsen D, et al (2010) Genetic fusions of heat-labile (LT) and heat-stable (ST) toxoids of porcine enterotoxigenic Escherichia coli elicit neutralizing anti-LT and anti-STa antibodies. Infection and immunity 78: 316–325. 19858307 10.1128/IAI.00497-09 PMC2798211

[42] Erickson AK, Willgohs JA, McFarland SY, Benfield DA, Francis DH (1992) Identification of two porcine brush border glycoproteins that bind the K88ac adhesin of Escherichia coli and correlation of these glycoproteins with the adhesive phenotype. Infection and immunity 60: 983–988. 1347288 10.1128/iai.60.3.983-988.1992 PMC257584

[43] Som P, Atkins HL, Bandoypadhyay D, Fowler JS, MacGregor RR, et al (1980) A fluorinated glucose analog, 2-fluoro-2-deoxy-D-glucose (F-18): nontoxic tracer for rapid tumor detection. J Nucl Med 21: 670–675. 7391842

[44] Nishikawa Y, Ogasawara J, Helander A, Haruki K (1999) An outbreak of gastroenteritis in Japan due to Escherichia coli O166. Emerging infectious diseases 5: 300. 10.3201/eid0502.990220 PMC2640687 10221888

[45] Nataro JP, Deng Y, Cookson S, Cravioto A, Savarino SJ, et al (1995) Heterogeneity of enteroaggregative Escherichia coli virulence demonstrated in volunteers. The Journal of infectious diseases 171: 465–468. 7844392 10.1093/infdis/171.2.465

[46] Bijlsma IG, de Nijs A, van der Meer C, Frik JF (1982) Different pig phenotypes affect adherence of Escherichia coli to jejunal brush borders by K88ab, K88ac, or K88ad antigen. Infection and immunity 37: 891–894. 6752028 10.1128/iai.37.3.891-894.1982 PMC347621

[47] Sellwood R, Gibbons RA, Jones GW, Rutter JM (1975) Adhesion of enteropathogenic Escherichia coli to pig intestinal brush borders: the existence of two pig phenotypes. Journal of medical microbiology 8: 405–411. 1100837 10.1099/00222615-8-3-405

[48] Zhang W, Robertson DC, Zhang C, Bai W, Zhao M, et al (2008) Escherichia coli constructs expressing human or porcine enterotoxins induce identical diarrheal diseases in a piglet infection model. Appl Environ Microbiol 74: 5832–5837. 18658289 10.1128/AEM.00893-08 PMC2547035

[49] McVeigh A, Fasano A, Scott DA, Jelacic S, Moseley SL, et al (2000) IS1414, an Escherichia coli insertion sequence with a heat-stable enterotoxin gene embedded in a transposase-like gene. Infection and immunity 68: 5710–5715. 10992475 10.1128/iai.68.10.5710-5715.2000 PMC101527

[50] Piva IC, Pereira AL, Ferraz LR, Silva RSN, Vieira AC, et al (2003) Virulence markers of enteroaggregative Escherichia coli isolated from children and adults with diarrhea in Brasilia, Brazil. Journal of clinical microbiology 41: 1827–1832. 12734212 10.1128/JCM.41.5.1827-1832.2003 PMC154701

[51] Greenberg DE, Jiang ZD, Steffen R, Verenker MP, DuPont HL (2002) Markers of inflammation in bacterial diarrhea among travelers, with a focus on enteroaggregative Escherichia coli pathogenicity. The Journal of infectious diseases 185: 944–949. 11920319 10.1086/339617

[52] Steiner TS, Lima AA, Nataro JP, Guerrant RL (1998) Enteroaggregative Escherichia coli produce intestinal inflammation and growth impairment and cause interleukin-8 release from intestinal epithelial cells. The Journal of infectious diseases 177: 88–96. 9419174 10.1086/513809

[53] Chaudhuri RR, Sebaihia M, Hobman JL, Webber MA, Leyton DL, et al (2010) Complete genome sequence and comparative metabolic profiling of the prototypical enteroaggregative Escherichia coli strain 042. PloS one 5: e8801. 20098708 10.1371/journal.pone.0008801 PMC2808357

[54] Yamamoto T, Wakisaka N, Sato F, Kato A (1997) Comparison of the nucleotide sequence of enteroaggregative Escherichia coli heat-stable enterotoxin 1 genes among diarrhea-associated Escherichia coli. FEMS microbiology letters 147: 89–95. 9037769 10.1111/j.1574-6968.1997.tb10225.x

